# Managing novel therapies and concomitant medications in chronic lymphocytic leukemia: key challenges

**DOI:** 10.3389/fphar.2024.1517972

**Published:** 2025-01-03

**Authors:** Sofija Kozarac, Jelena Ivanovic, Marko Mitrovic, Kristina Tomic Vujovic, Isidora Arsenovic, Nada Suvajdzic-Vukovic, Andrija Bogdanovic, Ana Vidovic, Milena Todorovic-Balint, Jelena Bila, Mirjana Mitrovic, Danijela Lekovic, Irena Djunic, Marijana Virijevic, Aleksandar Trivic, Jelena Micic, Darko Antic

**Affiliations:** ^1^ Clinic of Hematology, University Clinical Centre of Serbia, Belgrade, Serbia; ^2^ Faculty of Medicine, University of Belgrade, Belgrade, Serbia; ^3^ Clinic for Otorhinolaryngology and Maxillofacial Surgery, University Clinical Centre of Serbia, Belgrade, Serbia; ^4^ Clinic for Obstetrics and Gynecology, University Clinical Centre of Serbia, Belgrade, Serbia

**Keywords:** chronic lymphocytic leukemia, Bruton tyrosine kinase inhibitors, venetoclax, drug-drug interactions, comorbidities

## Abstract

The treatment of chronic lymphocytic leukemia (CLL) consists of the continuous use of Bruton tyrosine kinase inhibitors (BTKis) such as ibrutinib, acalabrutinib, zanubrutinib and pirtobrutinib, or Bcl-2 inhibitors, such as venetoclax. Overall survival (OS) and progression-free survival (PFS) of CLL patients are significantly improved with the use of these therapies. Adverse effects (AEs) that can occur during treatment and the presence of pre-existing comorbidities in patients can influence subsequent treatment outcomes and, consequently, OS and PFS. Managing these AEs, including cardiologic toxicity and infections (including fungal infections), as well as treating cardiovascular and other comorbidities, can be challenging due to potential drug interactions with the medications used for the management of AEs and comorbidities. Therefore, this review examined the key challenges associated with the concomitant use of novel CLL therapies and medications for managing comorbidities and AEs. This review aims to enhance and facilitate the management of patients with CLL.

## 1 Introduction

The treatment of chronic lymphocytic leukemia (CLL), most prevalent adult leukemia, both initial and relapse management, consists of the continuous use of Bruton tyrosine kinase (BTK) inhibitors (BTKis) or the time-limited use of a Bcl-2 inhibitor (venetoclax), either used alone or in combination with CD20^+^ monoclonal antibodies (mAb). Covalent BTKis in current use include ibrutinib (a first-generation BTKi), and second-generation BTKis - acalabrutinib and zanubrutinib (Kutsch et al., 2022; Wierda et al., 2022; Eichhorst et al., 2021; Eichhorst et al., 2024; Byrd et al., 2015; Barr et al., 2022; Byrd et al., 2021; Sharman et al., 2020; Ghia et al., 2022). Pirtobrutinib is a highly selective, non-covalent, reversible BTKi, that is indicated for treatment of relapsed/refractory CLL (Wierda et al., 2022; Eichhorst et al., 2024; Thompson and Tam, 2023). In the relapsed/refractory setting, the combination of the phosphoinositide 3-kinase (PI3K) inhibitor idelalisib and rituximab may be considered as a treatment option when other alternatives are unavailable or unsuitable for the patient (Wierda et al., 2022; Eichhorst et al., 2021; Sharman et al., 2019; Nair and Cheson, 2016; Gopal and Graf, 2016).

The use of these therapies has greatly enhanced overall survival (OS) and progression-free survival (PFS) in patients with CLL, even in those with high-risk features such as unmutated immunoglobulin heavy chain variable, *TP53* aberrations, or complex karyotype. Clinical trials have shown 5-year progression-free survival rates exceeding 60% for patients treated with these agents, and OS continues to improve for most patients (Barr et al., 2022; Ghia et al., 2022; Seymour et al., 2022; Sharman et al., 2022; Tam et al., 2022a). However, adverse effects (AEs) during treatment, along with pre-existing comorbidities, can compromise future treatment, OS, and PFS (Burger et al., 2015; Awan et al., 2019; Tam et al., 2022b; Itsara et al., 2023; Al-Sawaf et al., 2024; Fischer et al., 2019; Fresa et al., 2024). Along with BTK inhibition, BTKis can express off-target activity by binding other cysteine-containing kinases, leading to AEs (McMullen et al., 2014; Awan et al., 2020a; Bond and Woyach, 2019; Xiao et al., 2020). Most common AEs are fatigue, bruising, infection, neutropenia, thrombocytopenia, arthralgia, bleeding, hypertension, and heart rhythm abnormalities. A few patients develop AEs with higher grades (Eichhorst et al., 2021; Eichhorst et al., 2024) when additional medical treatment is indicated or following temporary or permanent drug discontinuation (Tam et al., 2022b; Bond and Woyach, 2019; Honigberg et al., 2010; Paydas, 2019; Coutre et al., 2019). The frequently observed AEs of venetoclax when combined with CD20^+^ mAb included cytopenia, infections, diarrhea, nausea, fatigue, with neutropenia and infections with the most frequent being grade 3 or 4 AEs (Al-Sawaf et al., 2024; Fischer et al., 2019; Eichhorst et al., 2023). Idelalisib most common AEs include diarrhea, pyrexia, and fatigue, with grade ≥3 diarrhea occurring exclusively in the idelalisib group in clinical trials. As serious AEs were reported pneumonia and febrile neutropenia, at comparable rates between idelalisib and placebo groups, leading to similar treatment discontinuation rates (Sharman et al., 2019; Coutré et al., 2015; Furman et al., 2014).

Due to potential drug-drug interactions (DDIs), treatment decisions (use of BTKi, venetoclax and idelalisib) require the thorough assessment of comorbidities and reassessment of concomitant treatment, which makes management of AEs challenging (Fresa et al., 2024; Paydas, 2019; de Jong et al., 2018; de Zwart et al., 2016). Therefore, this review examined the key challenges associated with the concomitant use of novel CLL therapies and treatments for managing comorbidities and AEs. This review aimed to enhance and facilitate the management of patients of CLL.

## 2 Mechanisms of drug-drug interactions in BTKis, Bcl-2 and PI3K inhibitors

DDIs refer to the ability of one drug to alter the pharmacological action or effect of another drug when administered simultaneously or consecutively, that can lead to enhanced, reduced, or modified therapeutic effects, posing potential risks to patient safety and treatment efficacy (Marengoni et al., 2014; Magro et al., 2021; Johnell and Klarin, 2007).

The primary metabolic and elimination pathways of ibrutinib are mediated by cytochrome P450 (CYP) 3A (CYP3A), an enzyme responsible for the metabolism of several other medications (Finnes et al., 2017; Fancher and Pappacena, 2020; Scheers et al., 2015). Although CYP2D6 contributes to ibrutinib metabolism, its role is clinically negligible, with no significant impact observed across CYP2D6 genotype variations (e.g., poor vs. extensive metabolizers) (Finnes et al., 2017; Scheers et al., 2015).

Given the critical role of CYP3A in ibrutinib metabolism, DDIs involving potent CYP3A inhibitors or inducers can significantly alter exposure to ibrutinib and the levels of its metabolites (de Jong et al., 2018; de Zwart et al., 2016). Based on the observation that ibrutinib concentrations can increase to 20-fold when co-administered with ketoconazole, a physiologically based pharmacokinetic model was developed to predict the DDI potential of mild/moderate CYP3A4 inhibitors and strong/moderate CYP3A4 inducers on ibrutinib levels (de Zwart et al., 2016). Ibrutinib exposure increases when administered with strong and moderate CYP3A4 inhibitors (e.g., ketoconazole, grapefruit juice, voriconazole, erythromycin, and diltiazem), and decreases in the presence of strong CYP3A4 inducers (e.g., rifampin) (de Jong et al., 2018; de Zwart et al., 2016; de Jong et al., 2015). The study concluded that strong CYP3A4 inhibitors and inducers should be avoided during treatment with ibrutinib because their significant impact on its pharmacokinetics could lead to AEs from increased drug exposure or reduced therapeutic efficacy due to decreased exposure (de Jong et al., 2018; de Zwart et al., 2016).

P-glycoprotein (P-gp) and breast cancer resistance protein (BCRP) contribute to the distribution of ibrutinib and its metabolites. Ibrutinib has the potential to inhibit P-gp and BCRP when administered at recommended doses, resulting in increased concentrations of oral P-gp and BCRP substrates (Fromm, 2004; Imbruvica. European Medicines Agency, 2024).

CYP3A4 and P-gp are both involved in the metabolism of anticoagulants, a potential for their interaction with ibrutinib exists, which may increase their plasma levels and, consequently, the risk of bleeding (Ganatra et al., 2018; Di Minno et al., 2017). Herbs and botanicals, e.g., St John’s wort, garlic oil, *Panax ginseng*, and *Ginkgo biloba*, as well as grapefruit juice alterate CYP3A4 enzyme activity and when taken with certain medication (e.g., CLL therapy) may lead to herb-drug interactions. Usage of products and suplements based on these herbs are contraindicated with BTKis and venetoclax (de Jong et al., 2018; Finnes et al., 2017; Gurley et al., 2005; Nicolussi et al., 2020).

Acalabrutinib significantly interacts with both CYP3A inducers and inhibitors. Acalabrutinib interacts with P-gp and BCRP, serving as a substrate for these transport proteins, and can potentially increase exposure to BCRP substrates by inhibiting BCRP activity in the intestine (Fancher and Pappacena, 2020). The solubility of acalabrutinib decreases at higher pH levels. Proton-pump inhibitors (PPIs) increase the pH level of the gastric mucosa, leading to a significant decrease in acalabrutinib bioavailability. Histamine-H2 receptor antagonists inhibit stomach acid production and increase gastric pH, thereby reducing the bioavailability of acalabrutinib. Staggered doses of histamine-H2 receptors and acalabrutinib was required to reduce DDIs and optimize therapeutic outcomes (Sharma et al., 2022; Pepin et al., 2019). However, newer formulation with pH independent release, acalabrutinib maleate tablet, reduces the impact of pH on acalabrutinib systemic exposure, enabling a wider ranges of patients to benefit from this treatment (Sharma et al., 2022).

Zanubrutinib metabolism is primarily mediated by the CYP3A isoforms. *In vitro* studies have indicated that zanubrutinib can reversibly inhibit CYP2C8, CYP2C9, and CYP2C19 in human liver microsomes, and can induce a two-fold or greater increase in the mRNA expression of CYP2B6, CYP2C8, CYP2C9, and CYP3A in human hepatocytes. According to a DDI study at clinically relevant concentrations, zanubrutinib exerted minimal to no impact on the activity of CYP2C9, BCRP, and P-gp. A decrease of less than 50% in systemic exposure of substrates sensitive to CYP3A and CYP2C19 was observed (Ou et al., 2021).

Pirtobrutinib is a CYP3A4 substrate, so it is recommended to avoid strong CYP3A4 inhibitors and inducers during pirtobrutinib therapy whenever possible (Pharmacist’s Application to Practice, 2023).

Venetoclax exhibits similar DDIs as BTKis owing to its predominant metabolism by CYP3A enzymes and as a substrate and inhibitor of both P-gp and BCRP (Fan et al., 2024). The total clearance of venetoclax from the plasma decreased by 19% when co-administered with moderate CYP3A inhibitors, and by up to 84% when co-administered with potent CYP3A inhibitors (Jones et al., 2016).

Strong CYP3A inducers, significantly reduce venetoclax plasma exposure (Fan et al., 2024). Population pharmacokinetics analysis has suggested that venetoclax can be administered without dose adjustment when combined with P-gp inhibitors, although a 50% dose reduction is generally recommended (Fan et al., 2024). During the venetoclax ramp-up period, DDIs with strong CYP3A4 and P-gp inhibitors can result in life-threatening complications by significantly increasing the risk of tumor lysis syndrome (TLS) and severe myelosuppression (e.g., neutropenia, thrombocytopenia, or anemia). To mitigate these risks, the use of strong CYP3A4 inhibitors should be avoided whenever possible. If their use is unavoidable, careful venetoclax dose adjustment, along with proactive TLS prevention measures and close monitoring, is essential to ensure patient safety and treatment efficacy (Fan et al., 2024; Tambaro and Wierda, 2020). Bile acid sequestrants (cholestyramine, cholesevelam, colestipol) can reduce the absorption of venetoclax, which may diminish its effectiveness, and their concurrent use with venetoclax should be avoided. If co-administration is necessary, venetoclax should be taken at least 6 h after the bile acid sequestrant to minimize this interaction (Janssens, 2024; AbbVie News Center, 2024).

Idelalisib is metabolized mostly by aldehyde oxidase and CYP3A. Idelalisib is not a sensitive substrate and can be given with CYP3A and P-gp inhibitors with caution for signs of idelalisib toxicity, but strong CYP3A and P-gp inducers should be avoided due to their potential to reduce drug concentrations. Use of CYP3A substrates and idelalisib should be avoided because its major metabolite (GS-563117) acts as moderate inhibitor of CYP3A (Nair and Cheson, 2016; Gopal and Graf, 2016; Jin et al., 2015a; Jin et al., 2015b; Ramanathan et al., 2016). Mechanism of DDIs and CLL treatment are summarized in Table 1.

**TABLE 1:** Mechanism of drug-drug interactions in CLL treatment.

CLL treatment	Mechanism of action	Potential cause of DDIs	Drugs with potential interactions
Ibrutinib	Covalent BTK inhibitor	Alteration of CYP3A4 metabolic pathway by inducers or inhibitors P-gp and BCRP inhibition Inhibition of downstream regulation of collagen and glycoprotein VI	CYP3A4 inhibitors (e.g., ketoconazole, grapefruit juice, voriconazole, erythromycin, and diltiazem), and CYP3A4 inducers (e.g., rifampin); herbs and botanicals (e.g., St John’s wort, garlic oil, *Panax ginseng*, and *Ginkgo biloba)* direct oral anticoagulants, amiodarone, verapamil, diltiazem, digoxin, antiplatelets
Acalabrutinib		Alteration of CYP3A4 metabolic pathway by inducers or inhibitors P-gp and BCRP inhibition Increase of pH level of gastric mucosae	CYP3A4 inhibitors and inducers direct oral anticoagulants, amiodarone, verapamil, diltiazem, digoxin proton pump inhibitors, histamine-H2 receptor antagonists
Zanubrutinib		Alteration of CYP3A4 metabolic pathway by inducers or inhibitors	CYP3A4 inhibitors and inducers
Pirtobrutinib	Non-covalent BTK inhibitor	Alteration of CYP3A4 metabolic pathway by inducers or inhibitors	CYP3A4 inhibitors and inducers
Venetoclax	BCL2 inhibitor	Alteration of CYP3A4 metabolic pathway by inducers or inhibitors P-gp and BCRP inhibition Reduction of absorption	CYP3A4 inhibitors and inducers direct oral anticoagulants, amiodarone, verapamil, diltiazem, digoxin Bile acid sequestrants (cholestyramine, cholesevelam, colestipol)
Idelalisib	PI3K inhibitor	Alteration of CYP3A4 metabolic pathway by inducers or inhibitors	CYP3A4 substrates, inhibitors and inducers

## 3 BTKis and PI3K inhibitors and anticoagulants

CLL is associated with reduced platelet aggregation and often thrombocytopenia. Most bleeding events in patients with CLL are mild (grade 1–2) and commonly present as spontaneous bruising, petechiae, or hematomas. Approximately 5% of patients may experience more severe bleeding events (grade 3 or higher) (Byrd et al., 2016; Rushworth et al., 2013).

Bleeding complications from BTKis stem from off-target kinase inhibition (e.g., inhibition of Tec protein tyrosin kinase), disrupted platelet activation, and weakened glycoprotein VI signaling. Ibrutinib can impair platelet activation, reduce the collagen response, and decrease adhesion to the von Willebrand Factor, which may contribute to bleeding. The exact mechanisms of ibrutinib-associated bleeding are unclear, but both the disease and treatment likely contribute to platelet dysfunction (Lipsky et al., 2015; Lee et al., 2017; Awan et al., 2020b). Acalabrutinib, zanubrutinib and pirtobrutinib clinical trials have shown lower rates of major bleeding than ibrutinib, as they are more selective BTKis (Sharman et al., 2020; Brown et al., 2022; Nicolson et al., 2018; Bye et al., 2022). Approximately 10%–12% of patients treated with ibrutinib develop atrial fibrillation (AF) and require anticoagulant therapy, which heightens the risk of grade 3–4 bleeding events (Galitzia et al., 2024). The decision to begin anticoagulant therapy for AF should be tailored for each patient, considering the evaluation of risk scores for bleeding and stroke risk (Hindricks et al., 2021). Data regarding the safety of combining targeted therapy for CLL with anticoagulants are limited. Most BTKi trials have prohibited the simultaneous use of vitamin K antagonists due to the high bleeding incidence observed in earlier studies (Burger et al., 2015; Chanan-Khan et al., 2016).

The recommended anticoagulant treatment for patients receiving ibrutinib is direct oral anticoagulants, which are easier to administer and have fewer drug interactions. Rivaroxaban, apixaban and edoxaban (Xa inhibitors) are preferred over dabigatran because they have fewer drug interactions (Paydas, 2019; Quartermaine et al., 2023; Tang et al., 2018; Thorp and Badoux, 2018).

Idelalisib requires caution when co-administered with direct oral anticoagulants that are sensitive to strong P-gp or CYP3A4 modulators (Di Minno et al., 2017; Ramanathan et al., 2016; Mar et al., 2022).

## 4 BTKis, Bcl-2 and PI3K inhibitors and antimicrobial therapies

### 4.1 Immune dysfunction and infection risk in CLL

Patients with CLL have severe dysfunctions of both the innate and adaptive immune systems, exposing them to a higher risk of infections. In CLL, dendritic cells remain immature, resulting in suboptimal T-cell activation due to insufficient interleukin (IL)-12 production. The monocyte-phagocytic system is compromised, leading to impaired production of reactive oxygen species and increasing susceptibility to bacterial infections. Natural killer (NK) cells have altered expression of the NKG2D co-receptor with reduced cytotoxic capabilities. Reduced levels of complement components impair the activity of both the classical and alternative pathways, compromising the ability to opsonize bacteria using the C3b complement component (Rivera and Ferrajoli, 2022; Maffei et al., 2013; Orsini et al., 2003; Naseraldeen et al., 2021). CLL B-cells exhibit a phenotype similar to that of regulatory B-cells, leading to the suppression of immune responses primarily through the secretion of the IL-10. T-cells are dysfunctional, accompanied by elevated levels of regulatory T-cells. Hypogammaglobulinemia, which is observed in 85% of patients, is one of the most important causes of immunodeficiency in CLL and typically involves a reduction in IgG and IgA levels. In early-stage CLL, only a single immunoglobulin class is usually diminished, whereas in advanced CLL, all immunoglobulin classes are affected (Forconi and Moss, 2015; Crassini et al., 2018).

Beyond its effects on CLL cells, BTK inhibition can impair NK cell function and reduce the production of pro-inflammatory cytokines, such as tumor necrosis factor-α and IL-1β, leading to diminished activity of macrophages and neutrophils. Treatment with ibrutinib lowers the levels of both CD4^+^ and CD8^+^ T-cells, with cell counts recovering after 6 months of therapy and reducing in tumor burden. However, effector memory CD4^+^ and CD8^+^ T-cells may continue to decline, even after 12 months of treatment (Bojarczuk et al., 2014; Kohrt et al., 2014; Mulder et al., 2021). In the clinical trials examining ibrutinib, the incidence of infections was as high as 80%, with upper respiratory tract and urinary infections being the most common (Coutre et al., 2019). Grade ≥3 infections occurred in 40% of the patients, with pneumonia being the most frequent (12%–17%). The most common fungal infections were caused by Aspergillus, Cryptococcus and *P. jirovecii* (Byrd et al., 2015; Coutre et al., 2019; Tey et al., 2023; Ruchlemer et al., 2019). Considering the lack of the data, CLL guidelines do not suggest administering *Pneumocystis jirovecii* pneumonia prophylaxis for every patients, but only in the patients at high-risk (i.e., patients with relapsed/refractory disease). The incidence of *Aspergillus fumigatus* infection is higher in the early phase of treatment and among patients who were receiving corticosteroids. In a recent cross-trial comparison, similar changes during BTK inhibition were observed with ibrutinib and acalabrutinib (Hallek et al., 2018; Frei et al., 2020).

Venetoclax may increase the risk of bacterial and fungal infections owing to neutropenia and a reduction in T, B and NK cell levels; however other studies have shown that it may partially counteract the immunosuppressive environment by restoring the function of NK cells (de Weerdt et al., 2019; Roberts et al., 2016; Stilgenbauer et al., 2016). Meta-analysis showed that the probability of an increased infection risk during venetoclax treatment was 71.2% (Prosty et al., 2023).

PI3K inhibition by idelalisib increases the risk of infections by impairing T and NK cell functions, including reduced T-cell-mediated cytotoxicity, diminished granzyme B and cytokine secretion, and decreased proliferation and cytotoxicity of NK cells (Rohrbacher et al., 2021; Martinelli et al., 2018).

### 4.2 Antimicrobial therapy and BTKis, Bcl-2 and PI3K inhibitors dose modifications

For short-time use (up to 7 days) of antibacterial (e.g., azithromycin, erythromycin and clarithromycin) or antifungal agents (e.g., ketoconazole, itraconazole and voriconazole) that are strong CYP3A inhibitors, temporarily discontinuation of ibrutinib may be necessary. If combined with moderate CYP3A4 inhibitors, the ibrutinib dose should be reduced to 280 mg/day, with further reductions in cases of strong inhibitors like voriconazole or posaconazole (de Zwart et al., 2016; Fancher and Pappacena, 2020; Bose et al., 2016). Similarly, acalabrutinib, zanubrutinib, and pirtobrutinib, which share similar metabolic pathways, require dose adjustments when co-administred with CYP3A inducers or inhibitors. (Fancher and Pappacena, 2020; Ou et al., 2021; Pharmacist’s Application to Practice, 2023).

Azole antifungal drugs should be avoided during the ramp-up phase of venetoclax treatment. Once a therapeutic dose of venetoclax is established, it should be reduced by 75% if concomitant drugs are indicated for infection. Certain weak inhibitors of CYP3A4 (ciprofloxacin and fluconazole), may significantly interact with Bcl-2 inhibitors; thus, a 50% dose reduction is recommended (Fan et al., 2024; Freise et al., 2017; Bhatnagar et al., 2021). Dose reduction of venetoclax by 50% is suggested when used concurrently with P-gp inhibitors (e.g., azithromycin). Therefore, the best approach would be to monitor venetoclax concentrations during concurrent treatment, as therapeutic efficacy significantly depends on serum drug levels (Fan et al., 2024; Freise et al., 2017; Agarwal et al., 2018).

Use of antimictrobial agents, such as erythromicin, that are CYP3A substrates should be avoided with idelalisib (Ramanathan et al., 2016; Cuneo et al., 2019).

## 5 BTKis, Bcl-2 and PI3K inhibitors and treatment of cardiovascular diseases

BTKis and venetoclax have been associated with an increased risk of cardiovascular events such as arrhythmias and hypertension, underscoring the importance of regular cardiovascular monitoring. Idelalisib is associated with cardiovascular AEs, though these are not common. Patients often present with additional cardiovascular risk factors, including older age, smoking, diabetes, and pre-existing hypertension. Effective management of these patients requires hematologist-cardiologist collaboration to adjust treatments and address cardiovascular risks (Sharman et al., 2019; Coutré et al., 2015; Quartermaine et al., 2023; Grewal et al., 2021; Fedele and Opat, 2024; Larsson et al., 2022).

### 5.1 BTKis and Bcl-2 inhibitors and antiarrhythmics

BTKis are associated with an increased risk of arrhythmias, particularly AF and ventricular arrhythmias. Although the precise mechanism remains unclear, potential factors include their effects on ion channels and inflammatory pathways via off-target activities (Xiao et al., 2020; Seymour et al., 2023; Dickerson et al., 2019; Archibald et al., 2021; Lampson et al., 2017). In contrast, venetoclax does not directly cause arrhythmias, but it can contribute indirectly through conditions like tumor lysis syndrome, which may lead to electrolyte imbalances that trigger arrhythmias (Anderson et al., 2024).

Drug interactions between novel CLL therapies and antiarrhythmic medications can be significant because both classes of drugs may influence the other’s metabolism and therapeutic efficacy. Drugs used to treat arrhythmias that inhibit the CYP3A4 enzyme (e.g., dronedarone, verapamil, and digoxin) can increase the concentrations of BTKis and BCL-2 inhibitors, potentially increasing the risk of AE (Mar et al., 2022; Asnani et al., 2017). Although amiodarone may not interact with ibrutinib as directly as dronedarone, it should still be used cautiously because of its complex interactions, which could potentially influence ibrutinib metabolism (Ganatra et al., 2018; Mar et al., 2022; Asnani et al., 2017). Sotalol, amiodarone and flecainide prolong the QT interval and, when used in conjunction with drugs that affect cardiovascular function, such as BTKis, can exacerbate this effect (Galitzia et al., 2024; Mar et al., 2022; Asnani et al., 2017).

Effective management of these risks requires close cardiovascular monitoring, seamless coordination between hematology and cardiology departments, and thorough patient education.

### 5.2 BTKis, Bcl-2 and PI3K inhibitors and antiplatelet drugs

Low-dose aspirin is widely used for cardiovascular disease (CVD) prevention in high-risk population. Studies have raised concerns about trend of overuse, particularly in those at low CVD risk, where the potential benefits of aspirin are outweighed by the risk of bleeding. In CLL patients, many may have been using aspirin without clear justification prior to their diagnosis. It is essential to reassess aspirin use before starting treatment. This evaluation should carefully balance the patient’s cardiovascular risk factors against the heightened risk of bleeding, ensuring aspirin is used only when the benefits clearly outweigh the risks (Luepker et al., 2021; Kim et al., 2019; Yoo et al., 2024).

Studies have shown that BTKis may impair platelet function by inhibiting the downstream regulation of collagen and glycoprotein VI. Concomitant use of antiplatelet agents (aspirin and clopidogrel) with BTKis increases the risk of major bleeding events (Mendez-Ruiz et al., 2023; Jones et al., 2017; Jiang et al., 2022). The frequency of major bleeding increases in BTKi-treated patients who receive antiplatelet therapy, with an incidence of 2.5% (Jones et al., 2017). Similarly, acalabrutinib has the potential to enhance bleeding risk when combined with antiplatelet medications, such as dipyridamole. Acalabrutinib and zanubrutinib has been associated with a lower bleeding risk than the other BTKis (Galitzia et al., 2024; Mendez-Ruiz et al., 2023; Jiang et al., 2022). In case that double antiplatelet therapy or combination of anticoagulation and antiplatelet therapy is required, alternative treatment to BTKis should be provided (Quartermaine et al., 2023). Venetoclax can lower platelet counts, significantly increasing the risk of bleeding and amplifying the effects of antiplatelet agents, such as ticagrelor (Galitzia et al., 2024). Idelalisib may exhibit antiplatelet effects and cause minor bleeding, given the role of PI3K in platelet activation, adhesion, and thrombus development (Barrachina et al., 2021).

### 5.3 BTKis and Bcl-2 inhibitors and antihypertensives

Hypertension is a recognized AE of BTKis that can emerge as a new condition during treatment, or BTKis can aggravate pre-existing hypertension (Quartermaine et al., 2023). Managing pre-existing hypertension during BTKi treatment presents a challenge because of the risk of developing severe hypertension (grade 3 or 4) as an AE, as well as potential DDIs with antihypertensive medications (Galitzia et al., 2024; Dickerson et al., 2019). DDIs between antihypertensive medications and CLL therapy (BTKis and venetoclax) mainly involve CYP3A4 metabolism, potentially affecting the effectiveness or increasing the side effects of CLL agents (Galitzia et al., 2024; Dickerson et al., 2019; Fernandez Turizo et al., 2024; Gordon et al., 2023). The use of non-dihydropyridine calcium channel blockers (diltiazem and nifedipine) should be avoided because of their major DDIs with BTKis (Quartermaine et al., 2023). Regular monitoring and potential dose adjustments are crucial for managing these interactions and ensuring effective treatment while minimizing side effect.

## 6 Recommendations on concurrent use of BTKis, Bcl-2 and PI3K inhibitors and concomitant medication

Given the potential for DDIs during the concurrent use of CLL treatments and other medications, it is essential to assess comorbidities and all concurrent medications before initiating CLL therapy. This evaluation helps to identify potential interactions and determine whether alternative treatments should be considered (Fresa et al., 2024; Paydas, 2019).

Attention should be focused on CYP3A4 inducers and inhibitors, as they can reduce efficacy and increase the incidence of AEs (Fresa et al., 2024; Paydas, 2019). Table 2 provides a list of CYP3A4 inducers and inhibitors (Liu et al., 2007).

**TABLE 2:** List of CYP3A4 inducers and inhibitors.

Inhibitors		Inducers	
Strong	Moderate	Strong	Moderate
Clarithromycin	Amiodarone	Carbamazepine	Cenobamate
Itraconazole	Cimetidine	Phenobarbital	Dipyrone
Ketoconazole	Diltiazem	Phenytoin	Efavirenz
Levoketoconazole	Dronedarone	Primidone	Eslicarbazepine
Lopinavir	Erythromycin	Rifampicin	Modafinil
Posaconazole	Fluconazole		Rifabutin
Voriconazole	Grapefruit juice		Rifapentine
Ritonavir	Isavuconazole		
	Verapamil		

Strong CYP3A4 inhibitors should be avoided during treatment with ibrutinib, acalabrutinib and zanubrutinib. If a strong interacting medication is required for a short duration (up to 7 days), temporary discontinuation of ibrutinib and acalabrutinib may be necessary. Combined with strong CYP3A inhibitors, zanubrutinib should be administered at 80 mg/day and pirtobrutinib requires dose reduction to 50 mg/day (Fancher and Pappacena, 2020; Ou et al., 2021; Pharmacist’s Application to Practice, 2023). When used in combination with moderate CYP3A4 inhibitors, the dose of ibrutinib should be reduced to 280 mg/day, with further reductions of up to 140 mg/day and 70 mg/day if necessary, and the dose of acalabrutinib should be reduced to 100 mg/day. No dose adjustment is required when ibrutinib and acalabrutinib are used alongside mild CYP3A4 inhibitors (de Zwart et al., 2016; Fancher and Pappacena, 2020; Bose et al., 2016). If a CYP3A4 inducer is unavoidable, pirtobrutinib dose should be increased to 300 mg daily for patients on 200 mg daily, or increased by 50 mg for those on 50 mg or 100 mg daily (Pharmacist’s Application to Practice, 2023). During venetoclax treatment, strong CYP3A4 inducers should be avoided or administered with a 75% venetoclax dose reduction, whereas moderate CYP3A4 inducers require a 50% dose reduction of venetoclax (Freise et al., 2017; Bhatnagar et al., 2021).

**FIGURE 1:**
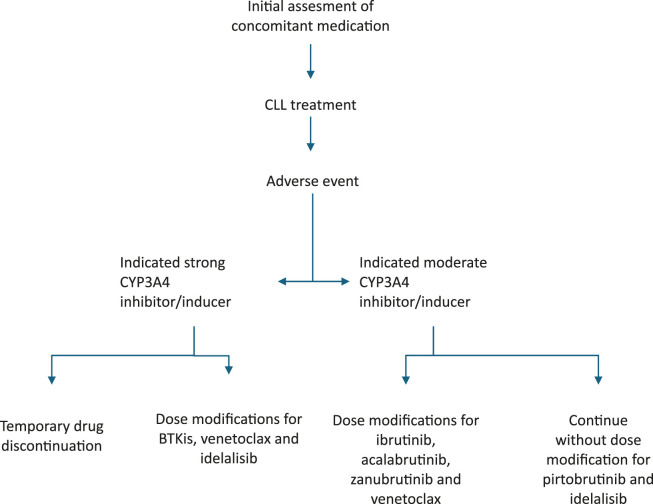
Suggested algorithm for assessment CLL treatment in case of CYP3A4 inhibitors/inducers treatment.

Table 3 provides dose adjustments for CLL therapy when co-administered with CYP3A4 inhibitors/inducers. Figure 1 present suggested algorithm for assessment CLL treatment in case of use of CYP3A4 inhibitors/inducers (Tam et al., 2022b; Al-Sawaf et al., 2024; Ramanathan et al., 2016; Awan et al., 2020b; Brown et al., 2022).

**TABLE 3:** Dose Adjustments for CLL therapy when co-administered with CYP3A4 inhibitors/inducers.

	Strong inducer/inhibitor	Moderate inducer/inhibitor
Ibrutinib	temporary discontinuation	280 mg/day
Acalabrutinib	temporary discontinuation	100 mg/day
Zanubrutinib	temporary discontinuation	80 mg/day
Pirtobrutinib	Inhibitor - 50 mg/day Inducer - dose reduction by 50 mg	Does not require reduction
Venetoclax	75% dose reduction	50% dose reduction
Idelalisib	temporary discontinuation	Does not require reduction

Owing to the increased risk of bleeding before and during ibrutinib treatment, the use of antiplatelet drugs should be reevaluated (Mendez-Ruiz et al., 2023; Jones et al., 2017). The preferred anticoagulant treatment for patients receiving ibrutinib is a direct oral anticoagulant (rivaroxaban, apixaban or edoxaban) (Paydas, 2019; Quartermaine et al., 2023).

## 7 Conclusion

To mitigate the risk of DDIs during the concurrent use of CLL treatment and other medications, it is crucial to thoroughly assess comorbidities and review all medications before initiating therapy. During treatment, any concomitant therapies used for managing AEs or comorbidities should be continuously reevaluated to ensure ongoing safety and effectiveness and to identify any emerging interactions that may necessitate dose adjustments in the treatment plan. Further studies are needed to provide more insight into the management of CLL in the context of concurrent medication use to offer a clearer comparison of the treatment options and their potential advantages and/or disadvantages. A multidisciplinary approach to treatment decisions when managing patients with CLL is crucial to ensuring optimal outcomes.
